# Iron status and dietary iron intake in relation to overweight/obesity in U.S. adults: a nationwide population-based study

**DOI:** 10.3389/fnut.2025.1617256

**Published:** 2025-10-09

**Authors:** Yuanyuan Lin, Yexin Chen, Jiangteng Liu, Minghao Li, Ying Tang, Jinxi Zhao, Yaofu Zhang

**Affiliations:** ^1^Dongzhimen Hospital, Beijing University of Chinese Medicine, Beijing, China; ^2^Guanganmen Hospital, China Academy of Chinese Medical Sciences, Beijing, China; ^3^Yuquan Hospital, Tsinghua University, Beijing, China

**Keywords:** iron, obesity, overweight, National Health and Nutrition Examination Survey (NHANES), transferrin saturation, cross-sectional study

## Abstract

**Background:**

Evidence on the associations between iron status biomarkers and both overweight/obesity prevalence and body mass index (BMI) is limited.

**Methods:**

This cross-sectional analysis utilized data from 5,454 participants in the NHANES 2003–2006 and 2017–2020 cycles. Overweight and obesity were defined as BMI ≥ 25 kg/m^2^ and ≥30 kg/m^2^, respectively. Weighted multivariable logistic and linear regression models were used to assess associations between iron biomarkers, dietary iron intake, and overweight/obesity risk or BMI. Restricted cubic splines (RCS) were utilized to explore potential non-linear patterns. Furthermore, subgroup analyses stratified by categorical covariates were conducted.

**Results:**

After adjusting for confounding variables, weighted logistic regression analysis identified reduced odds of overweight/obesity with higher dietary iron intake (OR = 0.98, *p* = 0.026), serum iron (SI; OR = 0.98, *p* = 0.004), and transferrin saturation (TSAT; OR = 0.98, *p* = 0.003). Weighted multivariable linear regression analysis demonstrated inverse associations of dietary iron intake (*β* = −0.06, *p* = 0.045), SI (*β* = −0.02, *p* < 0.001), and TSAT (*β* = −0.09, *p* < 0.001) with BMI. The total iron-binding capacity (TIBC) exhibited a marginal positive association with overweight/obesity risk and BMI. RCS analysis revealed non-linear dose–response relationships between SI, TSAT, TIBC, and overweight/obesity risk. After Bonferroni correction, no significant interaction effects were observed between iron biomarkers and stratified variables.

**Conclusion:**

Elevated dietary iron intake, serum iron, and TSAT are inversely associated with overweight/obesity risk, highlighting the potential protective role of adequate iron status in preventing obesity.

## Introduction

Overweight (defined as body mass index [BMI] ≥ 25 kg/m^2^) and obesity (BMI ≥ 30 kg/m^2^) affect over 2.11 billion adults globally, with U.S. age-standardized prevalence rates reaching 75.9% in men and 72.6% in women (1, 2). The disease burden linked to overweight and obesity is substantial, significantly elevating the risks of non-communicable diseases, including type 2 diabetes mellitus and cardiovascular disorders, as well as increasing susceptibility to over 13 types of cancer (3, 4). Most mortality related to non-communicable diseases, such as cancer, cardiovascular diseases, and respiratory diseases, reached its minimum within the BMI range of 21–25 kg/m^2^ (5). Research indicates that sedentary lifestyles, genetic susceptibility, and the globally prevalent Western dietary pattern—characterized by high calories (mainly from sugar and fats) yet deficiencies in micronutrients and fiber—are well-established contributors to obesity and diabetes (6, 7). Additionally, emerging evidence underscores the interplay between micronutrient imbalances and obesity pathogenesis, offering novel avenues for prevention and therapy (8).

Iron, as an indispensable trace element, plays a pivotal role in modulating metabolic rate, gluconeogenesis, insulin sensitivity, and adipocyte phenotype (9). Approximately 90% of the daily iron needs come from endogenous sources rather than dietary intake (10). Multiple biomarkers serve as indicators of iron status in individuals. Serum iron (SI) reflects the amount of iron bound to transferrin in the circulation, which is crucial for diagnosing iron deficiency and iron-overload conditions (11). Total iron-binding capacity (TIBC) quantifies the maximum amount of iron that can be bound by transferrin in serum, reflecting the body’s capacity to mobilize and deliver iron to tissues (12). Transferrin saturation (TSAT), calculated as the ratio of serum iron to TIBC, is a valuable indicator of iron availability for cells (12). Ferritin, the primary iron-storage protein in the body, functions as a key biomarker for assessing body iron reserves. It is critical to acknowledge, however, that ferritin serves as an acute-phase reactant, with its levels potentially elevated in inflammatory states, infections, or malignancies, irrespective of iron status (13). Iron status disorders may exacerbate obesity-related metabolic disorders by affecting mitochondrial function and cellular energy metabolism (14). Although a cross-sectional study of children aged 2 to 17 years suggested that higher dietary iron intake is associated with lower obesity risk (8), population-based evidence linking iron intake to adult obesity remains scarce. In addition, previous studies have reported conflicting findings regarding the association between iron status and overweight/obesity. A meta-analysis of 26 observational studies demonstrated lower serum iron and TSAT levels in overweight/obese individuals compared to normal-weight controls, with no significant difference in serum ferritin (15). In contrast, another study focusing on obese children found higher TIBC levels but no differences in serum iron or ferritin (16). Both studies were limited to group comparisons, and regression analyses to quantify associations, adjust for potential confounders, or explore dose–response relationships were lacking. Thus, the associations between dietary iron intake, iron status, and the risk of overweight/obesity in adults remain incompletely characterized.

Herein, utilizing data from the National Health and Nutrition Examination Survey (NHANES) database, this study simultaneously evaluated iron homeostasis biomarkers and dietary iron intake in relation to overweight/obesity risk and BMI, with the objective of providing evidence-based dietary insights for the prevention and management of adult obesity.

## Materials and methods

### Study population

This cross-sectional study used data derived from the NHANES. This nationally representative survey systematically collects comprehensive nutritional and health indicators of the American population through standardized physical examinations and interviews. The National Center for Health Statistics (NCHS) provides detailed documentation of NHANES study protocols, including standardized data collection manuals for questionnaires and laboratory tests, through its official portal at the Centers for Disease Control and Prevention.^1^ Ethical clearance for the NHANES survey protocol was granted by the NCHS Research Ethics Review Board. Prior to enrollment, documented informed consent forms were voluntarily signed by participants or legally authorized representatives (17).

**Figure 1:**
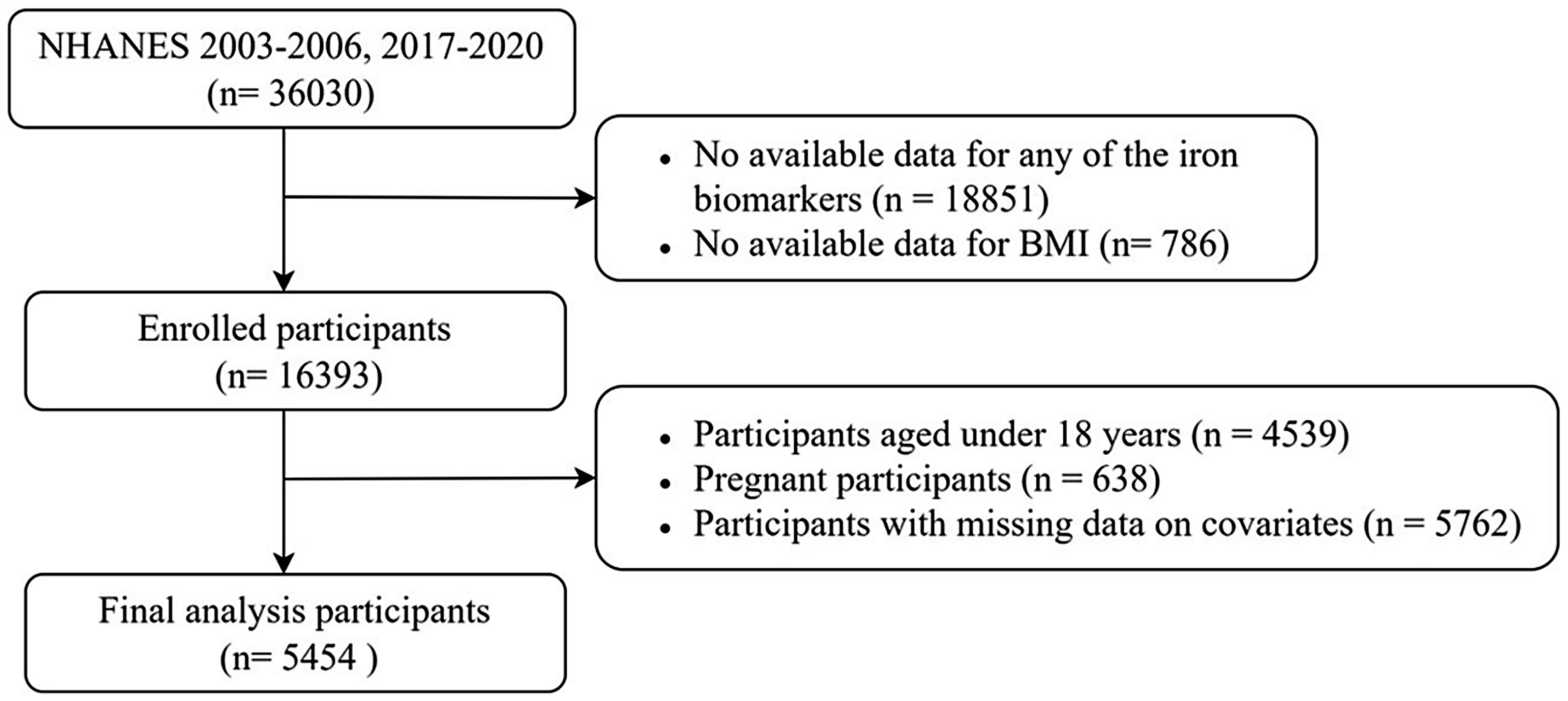
Flowchart of the study participants.

This study analyzed data from the NHANES 2003–2006 and 2017–2020 cycles. This selection was made to enhance the validity and generalizability of the statistical inferences, as these were the only cycles where data on both iron biomarkers and dietary iron intake were simultaneously available. The screening process is depicted in Figure 1. From an initial cohort of 36,030 participants, a total of 16,393 individuals with available data on at least one iron biomarker and BMI were enrolled. Subsequently, the study excluded 4,539 underage participants, 638 pregnant participants, and 5,762 participants with missing covariate data. As a result, a total of 5,454 participants were ultimately included in the final analysis. The complete-case analysis approach was adopted to avoid potential biases introduced by imputation methods.

### Exposure and outcome definitions

Exposure variables were defined as dietary iron intake and indicators of iron status, including serum iron, TIBC, TSAT, and ferritin. Dietary iron intake was assessed using a 24-h dietary recall survey. The initial dietary recall interview was conducted in-person at the Mobile Examination Center, and the second occurred by telephone 3 to 10 days later, administered by trained interviewers. Dietary intakes were calculated using the standardized United States Department of Agriculture (USDA) Food and Nutrient Database for Dietary Studies. The average of the two 24-h dietary recall information was used for analysis. In cases where participants lacked the second dietary interview, data from the first dietary recall were utilized. Serum iron was measured via a meticulous three-step procedure using the ferrozine reagent on the Roche Cobas 6,000 analyzer series. In this process, Fe^3+^ was liberated from transferrin, reduced to Fe^2+^, and complexed with ferrozine to form a colorimetric product, enabling its quantitative determination. TIBC was determined indirectly using measurements of serum iron and unsaturated iron-binding capacity obtained by the Roche method. TSAT was computed by dividing the measured value of frozen serum iron by the calculated TIBC and then multiplying the result by 100. Ferritin measurement was carried out using a sandwich immunoassay.

BMI was calculated as weight in kilograms divided by height in meters squared (kg/m^2^). Both height and weight were measured using a calibrated weight scale and a stadiometer by trained examiners and recorders. Adult weight status was classified according to the World Health Organization (WHO) classification criteria, where non-overweight/obesity was defined as a BMI of < 24.9 kg/m^2^, overweight as a BMI of ≥ 25 kg/m^2^, and obesity as a BMI of ≥ 30 kg/m^2^ (18).

### Covariates

Demographic and lifestyle factors used as covariates included age, sex, ethnicity, physical activity (PA), poverty–income ratio (PIR), and alcohol consumption. Physical activity was quantified as metabolic equivalent (MET) minutes of moderate-to-vigorous physical activity per week. Respondents were classified into three categories based on adult physical activity guidelines (19): highly active (≥1,200 MET-min/week, equivalent to ≥300 min/week of moderate-intensity or ≥150 min/week of vigorous-intensity activity), active (600–1,199 MET-min/week, equivalent to 150–299 min/week of moderate-intensity or 75–149 min/week of vigorous-intensity activity), and inactive (<600 MET-min/week). Poverty was measured using the PIR, which divided participants into low-income (PIR < 1.3) and high-income (PIR ≥ 1.3) categories. Alcohol consumption patterns were categorized into three distinct groups based on self-reported intake: non-drinkers were defined as individuals with no lifetime alcohol consumption; low-to-moderate drinkers, who consumed alcohol less than daily; and heavy drinkers, classified as individuals who consumed alcohol daily.

Clinical indicators linked to metabolic parameters were also included, such as diabetes status, total calorie intake, serum creatinine (SCR), alanine aminotransferase (ALT), aspartate aminotransferase (AST), total cholesterol (TC), and high-density lipoprotein cholesterol (HDL-C) (20–23). Diabetes mellitus was defined as a self-reported diagnosis via validated questionnaires or a glycosylated hemoglobin (HbA1c) level of ≥6.5%. The body roundness index (BRI), an indicator of visceral adiposity distribution, was calculated as: BRI = 364.2–365.5 × √[1 − (waist circumference/(2π))^2^/(0.5 × height)^2^], where waist circumference and height are in centimeters (24).

Given the critical role of dietary vitamin C intake in enhancing non-heme iron absorption and influencing iron metabolism (25), it was also included as a nutritional covariate. Total calorie intake was adjusted for, as it is a major determinant of body weight and may confound the association between iron status and obesity. Dietary vitamin C and calorie intake were quantified using 24-h dietary recall surveys. The analysis incorporated the mean of two 24-h dietary assessments. When participants lacked a second dietary recall, data from the first recall were used.

### Statistical analyses

To evaluate the differences in various variables across weight status groups, we employed the following statistical methods: continuous variables were presented as weighted median values with interquartile ranges (IQRs), while categorical variables were described using weighted frequencies and percentages. Group differences for continuous variables were assessed using the design-based Kruskal–Wallis test, which accounts for the complexities of the survey design and ensures the accuracy and reliability of the results. For categorical variables such as ethnicity, alcohol consumption, and physical activity, Pearson’s chi-squared test with Rao–Scott adjustment was used. This adjustment is designed to address potential biases due to the survey design, thereby making the chi-squared test results more robust and reliable.

Associations between iron status, dietary iron intake, and BMI were evaluated using weighted linear regression models. Weighted logistic regression models were used to examine the relationship between iron status, dietary iron intake, and the risk of overweight/obesity. To explore potential non-linear relationships between iron status, dietary iron intake, and the risk of obesity/overweight, we used restricted cubic spline (RCS) models with four knots positioned at the 5th, 35th, 65th, and 95th percentiles of the exposure distribution. This configuration was selected to balance the smoothness of the fitted curve and avoid loss of precision due to overfitting (26).

Furthermore, stratified multivariate regression was utilized for subgroup analyses, adjusting for all covariates except the stratification variable. We performed subgroup analyses stratified by categorical variables, including sex, ethnicity, PIR, physical activity, alcohol use, and diabetes status. To evaluate subgroup heterogeneity in associations, interaction terms were assessed via the likelihood ratio test (LRT). Specifically, LRT compared models with and without interaction terms (between exposure and stratifying covariates). A significant *p*-value for interaction indicated that the alternative model with interaction terms fitted the data better, confirming the presence of an interaction effect. All statistical tests were two-tailed, with statistical significance defined as a *p*-value of <0.05 for primary analyses (excluding subgroup analyses). For subgroup analyses involving multiple comparisons across stratified variables, the Bonferroni correction was applied to adjust the significance threshold: the original *α* = 0.05 was divided by the total number of independent subgroup tests (*n* = 6), resulting in a corrected significance level of α’ ≈ 0.008. All analyses were performed using R software (https://www.r-project.org/; version 4.4.2).

## Results

### Baseline characteristics of study participants

Among the 5,454 enrolled adults, 1,345 (25.51%) were categorized as non-overweight/non-obese, 1,655 (30.93%) as overweight, and 2,454 (43.56%) as obese. Table 1 presents the baseline characteristics and weighted estimates of adults with or without overweight/obesity in this study. Iron biomarkers differed significantly across weight categories: serum iron and TSAT decreased progressively with higher adiposity, from a median of 90.0 μg/dL and 29.00% in the non-overweight/obesity group to 79.0 μg/dL and 25.00% in the obesity group (*p* < 0.001), while ferritin increased from 80.20 ng/mL (IQR, 39.60–143.00) to 116.00 ng/mL (IQR, 57.00–198.00) (*p* < 0.001). TIBC, however, showed no statistically significant variation (*p* = 0.204). In addition, demographic and metabolic differences (sex, age, ethnicity, PIR, body roundness index, physical activity, and dietary vitamin C intake) were statistically significant across weight categories (All *p* < 0.001). Individuals with overweight/obesity exhibited a higher prevalence of diabetes, higher SCR, ALT, TC, and lower HDL-C. Nevertheless, no differences were observed in alcohol consumption, dietary iron intake, or caloric intake among these groups (all *p* > 0.05).

**Table 1:** Baseline characteristics of adults with or without overweight/obesity.

Characteristic	Non-obesity/overweight	Overweight	Obesity	*p*-value
	*N* = 1,345 (25.51%)	*N* = 1,655 (30.93%)	*N* = 2,454 (43.56%)	
Sex				0.026*
Male	13,293,886 (57.66%)	12,828,603 (45.88%)	20,019,755 (50.84%)	
Female	9,761,824 (42.34%)	15,133,599 (54.12%)	19,354,349 (49.16%)	
Age (years)	40.00 (26.00, 58.00)	51.00 (36.00, 66.00)	49.00 (34.00, 62.00)	<0.001*
Age group				<0.001*
18–29 years	7,486,991 (32.47%)	4,033,748 (14.43%)	6,502,013 (16.51%)	
30–49 years	7,004,994 (30.38%)	9,295,246 (33.24%)	13,757,170 (34.94%)	
50–69 years	6,297,666 (27.31%)	9,773,849 (34.95%)	14,197,788 (36.06%)	
70 years above	2,266,059 (9.83%)	4,859,359 (17.38%)	4,917,134 (12.49%)	
Ethnicity				<0.001*
Non-Hispanic White	15,350,896 (66.58%)	18,012,626 (64.42%)	24,985,754 (63.46%)	
Mexican American	1,205,652 (5.23%)	2,425,757 (8.68%)	3,919,101 (9.95%)	
Non-Hispanic Black	2,171,324 (9.42%)	2,443,406 (8.74%)	4,645,418 (11.80%)	
Other Hispanic	1,407,558 (6.11%)	2,272,703 (8.13%)	2,875,460 (7.30%)	
Other/multi-ethnicities	2,920,281 (12.67%)	2,807,709 (10.04%)	2,948,371 (7.49%)	
PIR				0.002*
<1.3	5,248,152 (22.76%)	4,539,746 (16.24%)	7,599,285 (19.30%)	
≥1.3	17,807,558 (77.24%)	23,422,455 (83.76%)	31,774,819 (80.70%)	
BRI	3.09 (2.54, 3.73)	4.74 (4.19, 5.46)	7.02 (6.04, 8.53)	<0.001*
BMI	22.50 (20.70, 23.70)	27.20 (26.20, 28.40)	34.10 (31.70, 38.30)	<0.001*
Alcohol use				0.069
Heavy	920,827 (3.99%)	1,129,090 (4.04%)	901,382 (2.29%)	
Low-to-moderate	19,978,717 (86.65%)	24,941,741 (89.20%)	36,140,938 (91.79%)	
Non-drinker	2,156,165 (9.35%)	1,891,370 (6.76%)	2,331,784 (5.92%)	
PA				0.009*
Active	2,703,853 (11.73%)	3,377,098 (12.08%)	3,896,495 (9.90%)	
Highly active	15,193,918 (65.90%)	16,983,160 (60.74%)	22,424,348 (56.95%)	
Inactive	5,157,940 (22.37%)	7,601,943 (27.19%)	13,053,261 (33.15%)	
Diabetes				<0.001*
Yes	1,110,626 (4.82%)	3,217,449 (11.51%)	8,034,109 (20.40%)	
No	21,945,084 (95.18%)	24,744,752 (88.49%)	31,339,995 (79.60%)	
Dietary VC intake	60.35 (29.20, 114.35)	60.05 (28.85, 103.25)	49.25 (24.70, 96.95)	0.002*
Dietary iron intake	12.46 (9.15, 16.60)	12.46 (9.26, 17.73)	12.47 (9.27, 16.27)	0.356
Total calorie intake	1,863.00 (1,471.00, 2,476.50)	1,968.50 (1,503.00, 2,485.00)	1,962.50 (1,536.00, 2,566.00)	0.216
Serum iron (μg/dL)	90.00 (69.00, 113.00)	89.00 (68.00, 111.00)	79.00 (60.00, 99.00)	<0.001*
TIBC (μg/dL)	316.00 (289.00, 355.00)	324.00 (292.00, 353.00)	324.00 (295.00, 354.00)	0.204
TSAT (%)	29.00 (22.00, 36.00)	28.00 (22.00, 35.00)	25.00 (18.00, 31.00)	<0.001*
Ferritin (ng/mL)	80.20 (39.60, 143.00)	110.00 (56.60, 190.00)	116.00 (57.00, 198.00)	<0.001*
HbA1c (%)	5.30 (5.10, 5.50)	5.50 (5.20, 5.80)	5.60 (5.30, 6.00)	<0.001*
SCR (umol/L)	70.72 (61.88, 82.21)	76.91 (65.42, 89.28)	75.14 (64.53, 87.52)	<0.001*
ALT (U/L)	15.00 (12.00, 20.00)	18.00 (13.00, 25.00)	21.00 (15.00, 30.00)	<0.001*
AST (U/L)	19.00 (16.00, 23.00)	19.00 (16.00, 24.00)	19.00 (16.00, 24.00)	0.396
TC (mmol/L)	4.55 (3.96, 5.22)	4.81 (4.19, 5.53)	4.78 (4.16, 5.51)	<0.001*
HDL-C (mmol/L)	1.55 (1.29, 1.81)	1.34 (1.11, 1.63)	1.19 (1.01, 1.42)	<0.001*

PIR, poverty–income ratio; BRI, body roundness index; BMI, body mass index; PA, physical activity; VC, vitamin C; TIBC, total iron-binding capacity; TSAT, transferrin saturation; SCR, serum creatinine; ALT, alanine aminotransferase; AST, aspartate aminotransferase; TC, total cholesterol; HDL-C, high-density lipoprotein cholesterol. * *p*-value < 0.05.

### Associations of iron status and adults with overweight/obesity

Multivariable-adjusted logistic regression models assessed the odds of overweight or obesity (BMI ≥ 25 kg/m^2^) in relation to iron biomarkers (Table 2). After completely adjusting for covariates, higher dietary iron intake was associated with 2% lower odds of overweight/obesity (OR = 0.98; 95% CI: 0.96–1.00; *p* = 0.026). Transferrin saturation demonstrated consistent inverse associations in three models, with each 1% increase corresponding to a 2% reduction in odds in model 3 (OR = 0.98; 95% CI: 0.96–0.99; *p* = 0.003). TIBC showed a marginal association (OR = 1.00; 95% CI: 0.99–1.00; *p* = 0.003), as the effect estimate and its confidence interval encompassed the null value. Serum iron also exhibited a non-significant inverse trend (OR = 1.00; 95% CI: 0.99–1.00; *P* = 0.004), while serum ferritin levels were unassociated with overweight/obesity (*P* > 0.05).

**Table 2:** Associations of iron status and dietary iron intake with the risk of overweight/obesity.

	Number	Model 1		Model 2		Model 3	
		OR (95%CI)	*p*-value	OR (95%CI)	*p*-value	OR (95%CI)	*p*-value
Iron intake (mg/day)	5,454	0.99 (0.98, 1.01)	0.374	0.98 (0.97, 1.00)	0.028*	0.98 (0.96, 1.00)	0.026*
Serum iron (μg/dL)	5,454	0.99 (0.99, 1.00)	<0.001*	0.99 (0.99, 1.00)	<0.001*	0.99 (0.99, 1.00)	0.004*
Transferrin saturation (%)	5,444	0.98 (0.97, 0.99)	<0.001*	0.98 (0.97, 0.98)	<0.001*	0.98 (0.96, 0.99)	0.003*
TIBC (μg/dL)	5,444	1.00 (1.00, 1.00)	0.202	1.00 (1.00, 1.01)	0.004*	1.00 (1.00, 1.01)	0.003*
Ferritin (ng/mL)	5,437	1.00 (1.00, 1.00)	0.002*	1.00 (1.00, 1.00)	0.025*	1.00 (1.00, 1.00)	0.504

Model 1: Non-adjusted.

Model 2: Adjusted for age, sex, ethnicity, and PIR.

Model 3: Adjusted for age, sex, ethnicity, PIR, PA, alcohol use, diabetes status, total calorie intake, vitamin C intake, SCR, ALT, AST, TC, and HDL-C.

* *p*-value < 0.05.

### Associations between iron status and iron intake with BMI

Multivariable linear regression analyses (Table 3) revealed significant inverse associations between serum iron and TSAT with BMI across all models, whereas TIBC demonstrated a positive association. These relationships persisted in the fully adjusted model (model 3): each 1 μg/dL increase in serum iron was associated with a 0.02-unit decrease in BMI (*β* = −0.02; 95% CI: −0.03 to −0.02; *p* < 0.001), and each 1% increase in TSAT corresponded to a 0.09-unit reduction in BMI (*β* = −0.09; 95% CI: −0.11 to −0.06; *p* < 0.001). In the linear regression analysis examining the association between TIBC and BMI, a marginally statistically significant positive association was observed (*β* = 0.01, 95% CI: 0.00 to 0.02; *p* = 0.022). Higher dietary iron intake was also linked to lower BMI in model 3 (*β* = −0.06; 95% CI: −0.12 to 0.00; *p* = 0.045). In contrast, no significant association was observed between serum ferritin levels and BMI (*P* > 0.05).

**Table 3:** Associations of iron status and dietary iron intake with BMI.

	Number	Model 1		Model 2		Model 3	
		*β* (95%CI)	*p*-value	*β* (95%CI)	*p*-value	*β* (95%CI)	*p*-value
Iron intake (mg/day)	5,454	−0.04 (−0.09, 0.01)	0.142	−0.03 (−0.08, 0.02)	0.215	−0.06 (−0.12, 0.00)	0.045*
Serum iron (μg/dL)	5,454	−0.04 (−0.04, −0.03)	<0.001*	−0.04 (−0.04, −0.03)	<0.001*	−0.02 (−0.03, −0.02)	<0.001*
Transferrin saturation (%)	5,444	−0.12 (−0.14, −0.10)	<0.001*	−0.12 (−0.14, −0.10)	<0.001*	−0.09 (−0.11, −0.06)	<0.001*
TIBC (μg/dL)	5,444	0.01 (0.00, 0.01)	0.013*	0.01 (0.00, 0.02)	0.008*	0.01 (0.00, 0.02)	0.022*
Ferritin (ng/mL)	5,437	0.00 (0.00, 0.00)	0.044*	0.00 (0.00, 0.01)	0.017*	0.00 (0.00, 0.00)	0.512

Model 1: Non-adjusted.

Model 2: Adjusted for age, sex, ethnicity, and PIR.

Model 3: Adjusted for age, sex, ethnicity, PIR, PA, alcohol use, diabetes status, total calorie intake, vitamin C intake, SCR, ALT, AST, TC, and HDL-C.

* *p*-value < 0.05.

### The non-linear relationship between iron status and overweight/obesity

**Figure 2:**
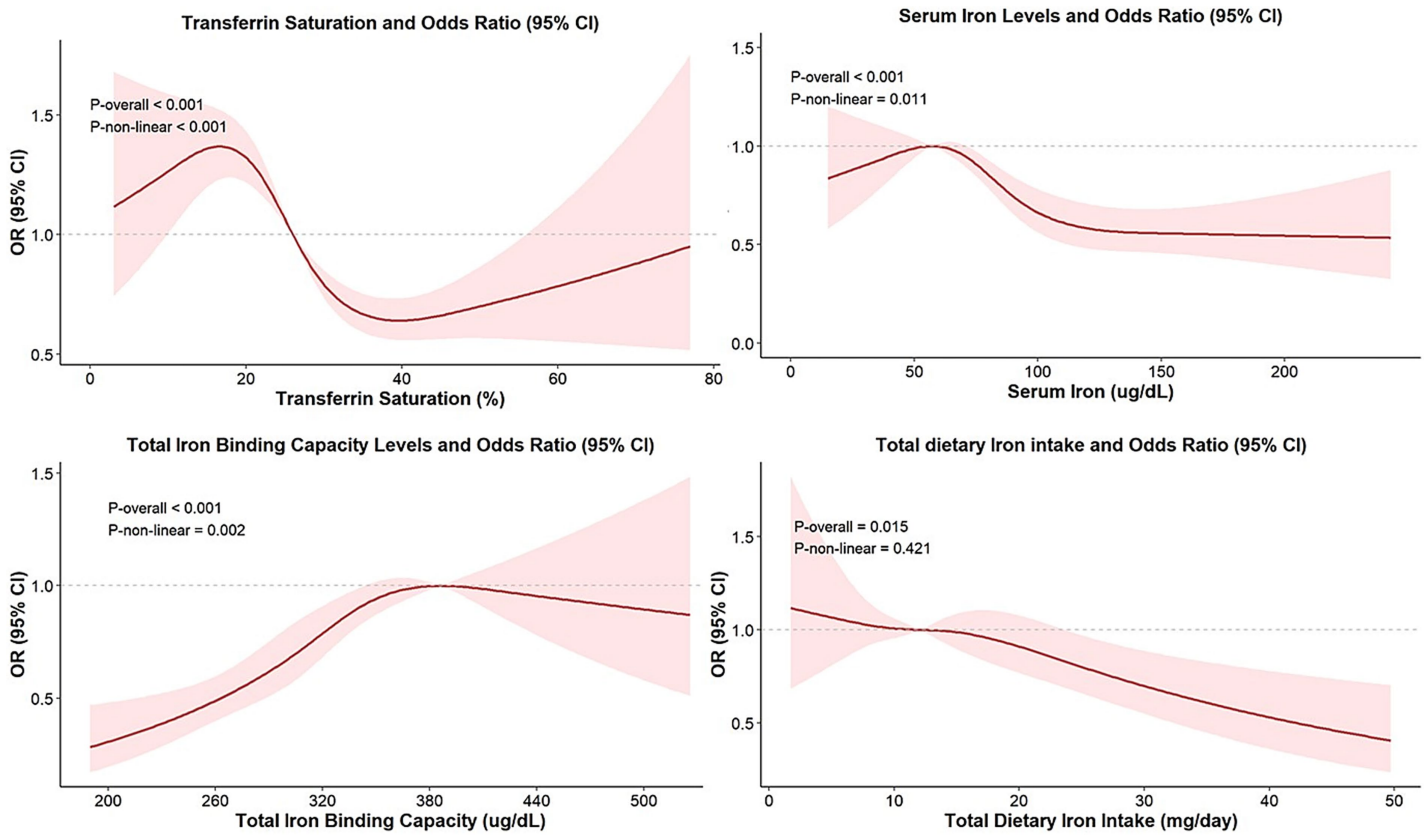
Non-linear relationship between iron status biomarkers and overweight/obesity. Models were adjusted for age, sex, ethnicity, PIR, PA, alcohol use, diabetes status, total calorie intake, vitamin C intake, SCR, ALT, AST, TC, and HDL-C.

As illustrated in Figure 2, non-linear associations between iron status biomarkers and overweight/obesity risk were evaluated using RCS regression models adjusted for age, sex, ethnicity, PIR, PA, alcohol use, diabetes status, total calorie intake, and vitamin C intake, SCR, ALT, AST, TC, and HDL-C. Notably, a threshold-dependent effect was observed, with a significant reduction in obesity risks when TSAT values ranged between 16.76 and 39.81% (*p*-overall < 0.001; *p*-non-linear < 0.001). An inverted U-shaped relationship was identified between SI and obesity risks (*p*-overall < 0.001; *p*-non-linear = 0.011), with the OR peaking at a turning point of 57.39 μg/dL. Beyond this level, the OR declined with increasing SI concentrations. Similarly, TIBC showed a non-linear trend (*p*-overall < 0.001; *p*-non-linear = 0.002), with the highest OR observed at approximately 385.86 μg/dL and decreasing risks thereafter. In addition, a continuously decreasing linear trend between total dietary iron intake and the prevalence of overweight/obesity was observed (*p*-overall < 0.015; *p*-non-linear = 0.421). No significant relationship was observed between ferritin and overweight/obesity (*p*-overall = 0.021; *p*-non-linear = 0.212).

### Subgroup analysis

Supplementary Tables 1, 2 present the results of subgroup analyses stratified by sex, ethnicity, PIR, PA, alcohol use, and diabetes status. After Bonferroni correction, no significant interaction effects were observed between iron biomarkers and stratified variables on either overweight/obesity risk or BMI in the multivariable-adjusted models (all interaction *p*-values > 0.008), indicating that the overall association pattern was consistent across the examined subgroups.

## Discussion

This study utilized data from the NHANES, a nationally representative cross-sectional dataset, to investigate the associations between iron status biomarkers and the risk of overweight/obesity, as well as BMI, among US adults. Specifically, overweight/obesity was negatively associated with dietary iron intake, serum iron levels, and transferrin saturation but positively associated with TIBC. Furthermore, these associations remained robust in the analysis of BMI: lower iron intake and transferrin saturation, alongside elevated TIBC, were significantly associated with higher BMI values.

Iron can alleviate obesity by regulating leptin levels and enhancing thermogenic capacity in adipose tissue. Serum leptin levels were observed to be 3.7-fold higher in obese individuals compared to their lean counterparts, while individuals with low iron status exhibited 3.2-fold higher serum leptin than those with high iron status (27). Hyperleptinemia-induced leptin resistance can contribute to obesity by disrupting hypothalamic satiety signaling (28). Mechanistically, iron has been shown to downregulate leptin transcription, with *in vitro* studies demonstrating iron-mediated suppression of leptin expression in adipocytes (27). Additionally, iron supplementation increased oxygen consumption and enhanced the thermogenic capacity of adipocytes, suggesting a promising approach to counteract obesity (29). Iron deficiency may also activate oxidative stress and elevate the expression of hypoxia-inducible factor 1 (HIF-1) (30). Overexpression of HIF-1*α* exacerbates adipose tissue dysfunction by inducing fibrosis and insulin resistance in white adipose tissue, thereby promoting obesity (31).

There is a bidirectional interplay between obesity and iron deficiency, wherein iron deficiency reciprocally promotes adiposity via dysregulated leptin levels and impaired adipose tissue function, while obesity impairs iron absorption through chronic inflammation and hepcidin-mediated disruption of iron transport. In individuals with obesity, pro-inflammatory cytokines such as interleukin-6 (IL-6), IL-1β, and tumor necrosis factor-*α* (TNF-α) can upregulate hepcidin, a central regulator of iron homeostasis (32, 33). This peptide promotes the degradation of the iron exporter ferroportin in macrophages, hepatocytes, and the duodenum, thereby inhibiting iron flow into the plasma, reducing iron absorption, and decreasing circulating iron availability (34, 35). Moreover, chronic exposure to TNF-α in intestinal cells can downregulate the expression of iron transporters, thereby impairing iron absorption and leading to iron deficiency (36).

TIBC reflects the total capacity of transferrin to bind iron, with elevated TIBC typically indicating a state of functional iron deficiency, wherein iron availability is limited despite normal or low iron stores. In our analysis, the persistent positive association between TIBC and BMI/obesity risk aligns with this physiological role: during iron-restricted states, the liver upregulates transferrin synthesis to enhance iron uptake as a compensatory mechanism, leading to elevated TIBC (37).

Heightened dietary iron intake may mitigate the risk of overweight/obesity and decrease BMI, suggesting that maintaining sufficient iron intake may serve as a protective nutritional strategy against excessive adiposity. Similarly, previous studies observed that higher levels of dietary iron intake were linked to a lower prevalence of overweight/obesity and metabolic syndrome in adolescents (38, 39). In the *Vitamin and Mineral Requirements in Human Nutrition, Second Edition,* formulated by the World Health Organization, the recommended daily iron intake for adult women is between 19.6 mg and 58.8 mg, while the recommended range for adult males is 9.1 mg to 27.4 mg (40). Given the reduced rate of iron absorption in patients with obesity and the protective effect of iron on obesity, adequate iron consumption within this range is recommended.

Ferritin, a key protein responsible for iron storage, sequesters excess iron to prevent oxidative cellular damage (41). In this study, we found no significant relationship between serum ferritin levels and overweight/obesity prevalence. Although elevated ferritin can reflect iron storage overload in healthy individuals, it may be elevated in the absence of hepatic iron overload in patients with obesity, metabolic syndrome, and alcoholism (42, 43). In inflammatory states, macrophages can secrete proinflammatory cytokines that stimulate ferritin synthesis, potentially decoupling ferritin from its classical role as iron status biomarkers (44). Elevated ferritin levels observed in this context should not be interpreted in isolation as evidence of iron overload, as they may primarily reflect the levels of inflammation associated with obesity and concomitant diseases rather than true iron excess (32, 43).

This study has several limitations. First, its cross-sectional design does not allow for causal inference. Future longitudinal or interventional studies are needed to clarify the causal direction between iron metabolism and obesity. Second, dietary iron intake was assessed via interview recall, which is prone to recall bias. Third, defining obesity solely based on the BMI is inadequate (45). Due to the diversity of the included population, multidimensional indicators such as waist circumference and body fat percentage were not incorporated. Future research should take these indicators into account to more precisely assess the individual health risks. Moreover, a potential limitation is that we were unable to uniformly include CRP or hsCRP as a covariate in sensitivity analyses across all cycles, due to methodological differences in the measurement of inflammatory markers. Although ferritin retains its core value as an indicator of iron stores, its interpretation may be influenced by inflammatory status (13). Thus, our analysis of ferritin’s association with overweight/obesity could not fully account for potential confounding by inflammation. Future studies should aim to more accurately isolate the independent effects of iron metabolism from those of inflammation.

## Conclusion

In conclusion, elevated levels of dietary iron intake, serum iron concentration, and transferrin saturation were linked to a lowered risk of overweight/obesity, suggesting that maintaining adequate iron status may play a crucial role in preventing and managing obesity. To further validate these associations, prospective cohort studies and mechanistic investigations are warranted to provide additional evidence and bolster this conclusion. Furthermore, evaluating iron deficiency in obese populations requires a comprehensive approach that incorporates multiple biomarkers to accurately assess iron status and inform targeted interventions.

## Data Availability

The original contributions presented in the study are included in the article/Supplementary material, further inquiries can be directed to the corresponding authors.

## Footnotes

^1^ https://www.cdc.gov/nchs/nhanes/
