# Supplementary material for: Iron status and dietary iron intake in relation to overweight/obesity in U.S. adults: a nationwide population-based study
Source: Front Nutr. 2025 Oct 9;12:1617256. doi: 10.3389/fnut.2025.1617256 (PMC12548546; doi:10.3389/fnut.2025.1617256)
Supplement: Supplementary file 1 [file Table_1.docx]

**Supplement**

**Table 1.** Subgroup analysis of the effect of iron status on overweight/obesity risk.

**Table 2.** Subgroup analysis of the effect of iron status on BMI.

**S****upplement table 1.** Subgroup analysis of the effect of iron status on overweight/obesity risk.

|  | Dietary Iron Intake (mg/day) | | Serum Iron (ug/dL) | | TIBC (ug/dL) | | Transferrin Saturation (%) | | Ferritin(ng/mL) | |
| --- | --- | --- | --- | --- | --- | --- | --- | --- | --- | --- |
|  | OR (95%CI) | *P* for interaction | OR (95%CI) | *P* for interaction | OR (95%CI) | *P* for interaction | OR (95%CI) | *P* for interaction | OR (95%CI) | *P* for interaction |
| Gender |  | 0.286 |  | 0.265 |  | 0.759 |  | 0.048 |  | 0.726 |
| Female | 0.99 (0.96, 1.02) |  | 0.99 (0.99, 1.00) |  | 1.00 (1.00, 1.01) |  | 0.97 (0.96, 0.99) |  | 1.00 (1.00, 1.00) |  |
| Male | 0.98 (0.96, 0.99) |  | 1.00 (0.99, 1.00) |  | 1.00 (1.00, 1.01) |  | 0.99 (0.98, 1.01) |  | 1.00 (1.00, 1.00) |  |
| Race and ethnicity |  | 0.589 |  | 0.765 |  | 0.450 |  | 0.888 |  | 0.401 |
| Non-Hispanic White | 0.99 (0.96, 1.01) |  | 0.99 (0.99, 1.00) |  | 1.00 (1.00, 1.01) |  | 0.98 (0.97, 1.00) |  | 1.00 (1.00, 1.00) |  |
| Mexican American | 0.98 (0.93, 1.03) |  | 1.00 (0.99, 1.00) |  | 1.00 (0.99, 1.00) |  | 0.99 (0.97, 1.02) |  | 1.00 (1.00, 1.00) |  |
| Non-Hispanic Black | 0.97 (0.95, 1.00) |  | 1.00 (0.99, 1.00) |  | 1.00 (1.00, 1.01) |  | 0.99 (0.97, 1.00) |  | 1.00 (1.00, 1.00) |  |
| Other Hispanic | 1.02 (0.95, 1.09) |  | 1.00 (0.99, 1.01) |  | 1.01 (1.00, 1.02) |  | 0.98 (0.95, 1.01) |  | 1.00 (1.00, 1.00) |  |
| Other/multiracial | 0.97 (0.94, 1.00) |  | 0.99 (0.99, 1.00) |  | 1.00 (1.00, 1.01) |  | 0.98 (0.96, 1.01) |  | 1.00 (1.00, 1.00) |  |
| PIR |  | 0.509 |  | 0.951 |  | 0.313 |  | 0.704 |  | 0.939 |
| < 1.3 | 1.02 (1.00, 1.05) |  | 1.00 (0.99, 1.01) |  | 1.00 (1.00, 1.01) |  | 0.98 (0.96, 1.01) |  | 1.00 (1.00, 1.00) |  |
| ≥ 1.3 | 0.97 (0.95, 0.99) |  | 0.99 (0.99, 1.00) |  | 1.00 (1.00, 1.01) |  | 0.98 (0.97, 1.00) |  | 1.00 (1.00, 1.00) |  |
| PA |  | 0.923 |  | 0.488 |  | 0.728 |  | 0.509 |  | 0.229 |
| Inactive | 0.96 (0.92, 1.00) |  | 1.00 (0.99, 1.00) |  | 1.00 (1.00, 1.01) |  | 0.98 (0.96, 1.00) |  | 1.00 (1.00, 1.00) |  |
| Active | 0.98 (0.92, 1.04) |  | 0.99 (0.98, 1.00) |  | 1.00 (0.99, 1.01) |  | 0.97 (0.95, 1.00) |  | 1.00 (1.00, 1.00) |  |
| High active | 0.99 (0.97, 1.01) |  | 0.99 (0.99, 1.00) |  | 1.00 (1.00, 1.01) |  | 0.98 (0.97, 1.00) |  | 1.00 (1.00, 1.00) |  |
| Alcohol use |  | 0.089 |  | 0.978 |  | 0.567 |  | 0.955 |  | 0.575 |
| Heavy | 0.89 (0.80, 0.99) |  | 0.99 (0.97, 1.01) |  | 1.00 (0.99, 1.01) |  | 0.96 (0.91, 1.01) |  | 1.00 (1.00, 1.00) |  |
| Low-to-moderate | 0.99 (0.96, 1.01) |  | 0.99 (0.99, 1.00) |  | 1.00 (1.00, 1.01) |  | 0.98 (0.97, 0.99) |  | 1.00 (1.00, 1.00) |  |
| Nondrinker | 1.02 (0.95, 1.10) |  | 1.00 (0.99, 1.01) |  | 1.00 (0.99, 1.01) |  | 0.99 (0.97, 1.02) |  | 1.00 (1.00, 1.01) |  |
| Diabetes |  | 0.945 |  | 0.584 |  | 0.154 |  | 0.524 |  | 0.462 |
| Yes | 0.96 (0.91, 1.02) |  | 1.00 (0.99, 1.01) |  | 1.00 (0.99, 1.00) |  | 1.01 (0.97, 1.04) |  | 1.00 (1.00, 1.00) |  |
| No | 0.99 (0.97, 1.00) |  | 0.99 (0.99, 1.00) |  | 1.00 (1.00, 1.01) |  | 0.98 (0.97, 0.99) |  | 1.00 (1.00, 1.00) |  |

Model was adjusted for age, gender, race, PIR, PA, alcohol use, diabetes, total calories intake, vitamin C intake, SCR, ALT, AST, TC and HDL-C.

* *p*-value＜0.008. Bonferroni correction was applied for multiple testing.

**Supplement** **table 2.** Subgroup analysis of the effect of iron status on BMI.

|  | Dietary Iron Intake (mg/day) | | Serum Iron (ug/dL) | | TIBC (ug/dL) | | Transferrin Saturation (%) | | Ferritin(ng/mL) | |
| --- | --- | --- | --- | --- | --- | --- | --- | --- | --- | --- |
|  | β (95%CI) | *P* for interaction | β (95%CI) | *P* for interaction | β (95%CI) | *P* for interaction | β (95%CI) | *P* for interaction | β (95%CI) | *P* for interaction |
| Gender |  | 0.686 |  | 0.457 |  | 0.024 |  | 0.067 |  | 0.971 |
| Female | -0.07 (-0.19, 0.05) |  | -0.03 (-0.04, -0.02) |  | 0.02 (0.01, 0.02) |  | -0.11 (-0.14, -0.07) |  | 0.00 (0.00, 0.00) |  |
| Male | -0.05 (-0.11, 0.01) |  | -0.02 (-0.03, -0.01) |  | 0.00 (-0.01, 0.01) |  | -0.06 (-0.08, -0.04) |  | 0.00 (0.00, 0.00) |  |
| Race and ethnicity |  | 0.241 |  | 0.563 |  | 0.342 |  | 0.491 |  | 0.299 |
| Non-Hispanic White | -0.09 (-0.14, -0.03) |  | -0.03 (-0.03, -0.02) |  | 0.01 (0.00, 0.02) |  | -0.09 (-0.12, -0.06) |  | 0.00 (0.00, 0.00) |  |
| Mexican American | -0.04 (-0.13, 0.05) |  | -0.03 (-0.05, -0.01) |  | -0.00 (-0.01, 0.01) |  | -0.09 (-0.16, -0.02) |  | 0.00 (0.00, 0.00) |  |
| Non-Hispanic Black | -0.07 (-0.16, 0.02) |  | -0.02 (-0.04, 0.00) |  | 0.01 (-0.00, 0.02) |  | -0.07 (-0.12, -0.02) |  | 0.00 (0.00, 0.00) |  |
| Other Hispanic | 0.15 (-0.08, 0.38) |  | -0.01 (-0.04, 0.01) |  | 0.02 (0.00, 0.03) |  | -0.05 (-0.13, 0.02) |  | 0.00 (-0.01, 0.00) |  |
| Other/multiracial | -0.00 (-0.08, 0.08) |  | -0.02 (-0.04, 0.00) |  | 0.00 (-0.01, 0.01) |  | -0.06 (-0.12, -0.01) |  | 0.00 (0.00, 0.00) |  |
| PIR |  | 0.481 |  | 0.167 |  | 0.305 |  | 0.077 |  | 0.930 |
| < 1.3 | 0.01 (-0.07, 0.09) |  | -0.04 (-0.05, -0.02) |  | 0.01 (0.00, 0.02) |  | -0.13 (-0.18, -0.08) |  | 0.00 (0.00, 0.01) |  |
| ≥ 1.3 | -0.09 (-0.15, -0.02) |  | -0.02 (-0.03, -0.02) |  | 0.01 (0.00, 0.02) |  | -0.07 (-0.09, -0.05) |  | 0.00 (0.00, 0.00) |  |
| PA |  | 0.070 |  | 0.486 |  | 0.111 |  | 0.576 |  | 0.299 |
| Inactive | 0.01 (-0.11, 0.14) |  | -0.02 (-0.03, -0.00) |  | 0.02 (0.01, 0.03) |  | -0.08 (-0.12, -0.03) |  | 0.00 (0.00, 0.00) |  |
| Active | 0.05 (-0.05, 0.15) |  | -0.02 (-0.04, -0.00) |  | -0.00 (-0.02, 0.01) |  | -0.06 (-0.12, -0.01) |  | 0.00 (0.00, 0.01) |  |
| High active | -0.09 (-0.14, -0.04) |  | -0.03 (-0.03, -0.03) |  | 0.01 (0.00, 0.02) |  | -0.09 (-0.11, -0.08) |  | 0.00 (0.00, 0.00) |  |
| Alcohol use |  | 0.458 |  | 0.353 |  | 0.197 |  | 0.247 |  | 0.392 |
| Heavy | -0.12 (-0.22, -0.02) |  | -0.02 (-0.04, -0.00) |  | -0.00 (-0.02, 0.02) |  | -0.05 (-0.13, 0.04) |  | 0.00 (0.00, 0.01) |  |
| Low-to-moderate | -0.05 (-0.10, -0.00) |  | -0.02 (-0.03, -0.02) |  | 0.01 (0.01, 0.02) |  | -0.09 (-0.10, -0.07) |  | 0.00 (0.00, 0.00) |  |
| Nondrinker | -0.08 (-0.25, 0.10) |  | -0.03 (-0.06, -0.01) |  | -0.00 (-0.02, 0.01) |  | -0.09 (-0.16, -0.02) |  | 0.00 (0.00, 0.01) |  |
| Diabetes |  | 0.503 |  | 0.852 |  | 0.423 |  | 0.530 |  | 0.027 |
| Yes | -0.14 (-0.27, -0.01) |  | -0.02 (-0.04, 0.00) |  | 0.01 (0.00, 0.02) |  | -0.08 (-0.13, -0.03) |  | 0.00 (0.00, 0.00) |  |
| No | -0.05 (-0.10, 0.01) |  | -0.03 (-0.03, -0.02) |  | 0.01 (0.00, 0.02) |  | -0.09 (-0.10, -0.07) |  | 0.00 (0.00, 0.00) |  |

Model was adjusted for age, gender, race, PIR, PA, alcohol use, diabetes, total calories intake, vitamin C intake, SCR, ALT, AST, TC and HDL-C.

* *p*-value＜0.008. Bonferroni correction was applied for multiple testing.
